# Offset Responses in the Auditory Cortex Show Unique History Dependence

**DOI:** 10.1523/JNEUROSCI.0494-22.2022

**Published:** 2022-09-28

**Authors:** Timothy Olsen, Andrea R. Hasenstaub

**Affiliations:** ^1^Coleman Memorial Laboratory; ^2^Department of Otolaryngology–Head and Neck Surgery, University of California, San Francisco, California 94143

**Keywords:** auditory cortex, history dependence, interneuron, offset, plasticity, temporal

## Abstract

Sensory responses typically vary depending on the recent history of sensory experience. This is essential for processes, including adaptation, efficient coding, and change detection. In the auditory cortex (AC), the short-term history dependence of sound-evoked (onset) responses has been well characterized. Yet many AC neurons also respond to sound terminations, and little is known about the history dependence of these “offset” responses, whether the short-term dynamics of onset and offset responses are correlated, or how these properties are distributed among cell types. Here we presented awake male and female mice with repeating noise burst stimuli while recording single-unit activity from primary AC. We identified parvalbumin and somatostatin interneurons through optotagging, and also separated narrow-spiking from broad-spiking units. We found that offset responses are typically less depressive than onset responses, and this result was robust to a variety of stimulus parameters, controls, measurement types, and selection criteria. Whether a cell's onset response facilitates or depresses does not predict whether its offset response facilitates or depresses. Cell types differed in the dynamics of their onset responses, and in the prevalence, but not the dynamics, of their offset responses. Finally, we clustered cells according to spiking responses and found that response clusters were associated with cell type. Each cluster contained cells of several types, but even within a cluster, cells often showed cell type-specific response dynamics. We conclude that onset and offset responses are differentially influenced by recent sound history, and discuss the implications of this for the encoding of ongoing sound stimuli.

**SIGNIFICANCE STATEMENT** Sensory neuron responses depend on stimulus history. This history dependence is crucial for sensory processing, is precisely controlled at individual synapses and circuits, and is adaptive to the specific requirements of different sensory systems. In the auditory cortex, neurons respond to sound cessation as well as to sound itself, but how history dependence is used along this separate, “offset” information stream is unknown. We show that offset responses are more facilitatory than sound responses, even in neurons where sound responses depress. In contrast to sound onset responses, offset responses are absent in many cells, are relatively homogeneous, and show no cell type-specific differences in history dependence. Offset responses thus show unique response dynamics, suggesting their unique functions.

## Introduction

A universal feature of sensory processing is that the brain's response to a sensory stimulus depends on the recent history of sensory experience. This history dependence can take many forms: depending on the stimulus, brain area, and delay, one stimulus might increase or reduce responses to subsequent, similar stimuli. History-dependent increases and decreases in firing have different implications for how sensory stimuli are processed, and likely relate to differences in functional requirements. For example, for many cells in the auditory system, a repeated pure tone will reduce (or “depress”) responses to subsequent, similar tones, which likely enhances representation of novel sounds (Nelken, 2014). In others, sound stimuli can increase (or “facilitate”) responses to a subsequent sound stimulus, thus enhancing the representation of sound sequences (Brosch and Schreiner, 2000).

In the auditory cortex (AC), in addition to responding (mostly transiently) to the onset of sound, some neurons respond to the ends (or “offsets”) of sounds. Much is known about the history dependence of cortical sound “onset” responses, including the relative tendencies of responses to facilitate or depress (depression predominates), the time constants of facilitation or depression, and the relationship between cell type and facilitation or depression (Brosch and Schreiner, 1997; Ulanovsky et al., 2004; Wehr and Zador, 2005). Yet almost nothing is known about the history dependence of sound offset responses. Sound offset responses are important for many aspects of hearing, which are key to processing continuous sounds, such as speech (Liberman et al., 1954; Ghitza, 2011), including the encoding of sound duration (Malone et al., 2015; Li et al., 2021; Sołyga and Barkat, 2021), the discrimination of up versus down frequency-modulated (FM) sweeps (Sollini et al., 2018), and the detection of silent gaps in between sounds (Anderson and Linden, 2016; Weible et al., 2020). Thus, understanding how offset responses depend on recent stimulus history is likely to aid in our understanding of how the brain processes complex, naturalistic sounds.

Several aspects of AC circuitry support the prediction that onset and offset responses will be differently affected by recent sound history. The input pathways driving onset and offset responses are distinct and largely nonoverlapping (He, 2001; Scholl et al., 2010; Liu et al., 2019), which could allow differences in history dependence to be inherited from differences in the dynamics of their thalamic inputs. In addition, the many types of cortical interneurons may shape history dependence in diverse and specific ways. Prior work has shown that parvalbumin-positive (PV) and somatostatin-positive (SST) interneurons control separate types of history dependence in the cortex (Natan et al., 2015; Phillips et al., 2017), in part because of specializations in their own short-term synaptic dynamics. Because the types of cortical interneurons that tend to show onset versus offset responses are different (Liu et al., 2019; Li et al., 2021), this implies that cortical interneurons may shape onset and offset history dependence in distinct ways.

Here, we compare the prevalence of history-dependent facilitation and depression in offset and onset responses, both across populations of cortical neurons of different excitatory and inhibitory types and within individual neurons. We presented awake mice with a series of repeating noise bursts and silent intervals, recorded through layers of primary AC with a silicon probe, and measured the dynamics of onset and offset responses over sound repetitions. We separately tracked responses in opto-tagged populations of PV and SST interneurons and in untagged broad-spiking (putative pyramidal) and narrow-spiking (nonpyramidal) cells. We show that offset response magnitudes do depend on recent stimulus history and that they are less likely to depress than onset responses. Different cell types have different proportions of onset- and offset-responsive cells, and their onset responses, but not their offset responses, show diverse forms of history dependence. Last, we show that, while early onset firing tended to depress, later or more sustained firing was more facilitatory; in contrast, nearly all offset responses were transient, and there was almost no relationship between offset response duration, cell type, and history dependence.

## Materials and Methods

### Subjects

All procedures were approved by the Institutional Animal Care and Use Committee at the University of California, San Francisco. Across all experiments, 23 male and female adult mice served as experimental subjects (median age: 80 d, range: 49-142 d). To target PV^+^ or SST^+^ cells for optogenetic manipulation, we crossed female PV-Cre (Jax strain: 012358) or SST-Cre (Jax strain: 013044) knockin mouse lines with male Ai32 (Cre-dependent ChR2) mice (Jax strain: 012569). All Cre lines were on a C57/Bl6J background. Animals were housed in cages of 2-5 under a 12 h-12 h light-dark cycle. Food and water were provided *ad libitum*.

### Surgical preparation

An initial surgery under isoflurane anesthesia (4% for induction, 1%-2% for maintenance) was performed to fix a custom metal headbar to the right temporal skull with dental cement. After at least 2 d of recovery, mice were reanesthetized using isoflurane, dexamethasone (4 mg/kg) was administered to reduce swelling, and a ∼1-2 mm craniotomy was made under the squamosal ridge at a location ∼2.0–3.0 mm posterior to bregma to expose the AC. Following the craniotomy, the animal was allowed to recover for at least 2 h before electrophysiological recordings began. During recordings, the craniotomy site was filled with agarose (2%) to prevent the brain from being exposed to the environment. When not performing recordings, craniotomies were covered with agarose (2%) and subsequently sealed with silicone elastomer (Kwik-cast, World Precision Instruments). Recordings were performed daily for up to 6 d after surgery.

### Electrophysiology

We performed all recordings inside a sound attenuation chamber (Industrial Acoustics). Mice were head-fixed and placed on a spherical treadmill (Phillips et al., 2017), and a 64 channel, linear silicon probe was inserted into the AC (Cambridge Neurotech, model H9: single shank with a staggered electrode channel formation: 45 μm vertical spacing, 22.5 μm horizontal spacing). The distance from the shallowest to deepest channel was 1417 μm, which allowed us to record across and beyond the entire span of the cortex (∼800 μm) (Paxinos and Franklin, 2019). The probe was continuously lowered into the brain at an angle perpendicular to the brain surface until we observed silent channels both superficial (corresponding to the agarose above the brain) and deep (putative white matter), typically 900-1000 μm. In some experiments, an optical fiber (Thor Labs, 400 μm tip diameter, 0.39 NA) was placed directly above the surface of the brain, parallel to the recording electrode. We then covered the craniotomy with agarose (2%) and let the probe settle for ≥30 min before recordings began. Throughout the experiment, extracellular voltage recordings were collected continuously at a sampling rate of 24,414 Hz using a 64-channel TDT-RZ2 system and Synapse software (Tucker-Davis Technologies). Multiunit activity was obtained from the extracellular voltage recordings following bandpass filtering of the voltage signal at 300-6000 Hz (filtering performed in the forward and reverse direction using a fourth-order elliptical filter). Negative threshold crossings exceeding 5 median absolute deviations from baseline were considered multiunit activity (Rey et al., 2015). We obtained single-unit activity by spike sorting the extracellular voltage recordings using Kilosort 2.0 (Pachitariu et al., 2016), and manually validating the spike sorting using Phy (https://github.com/cortex-lab/phy).

### Sound presentation

Sound stimuli were generated using MATLAB (The MathWorks) and delivered through a free-field electrostatic speaker (ES1, Tucker-Davis Technologies) driven by an internal soundcard running at 192 kHz. The speaker was placed ∼20 cm away from the mouse and pointed at the ear contralateral to the recording site. Sound levels were calibrated to 60 ± 5 dB at ear position (using a Brüel & Kjær 2209 and model 4939 microphone).

### Sound stimuli

**Figure 1.**
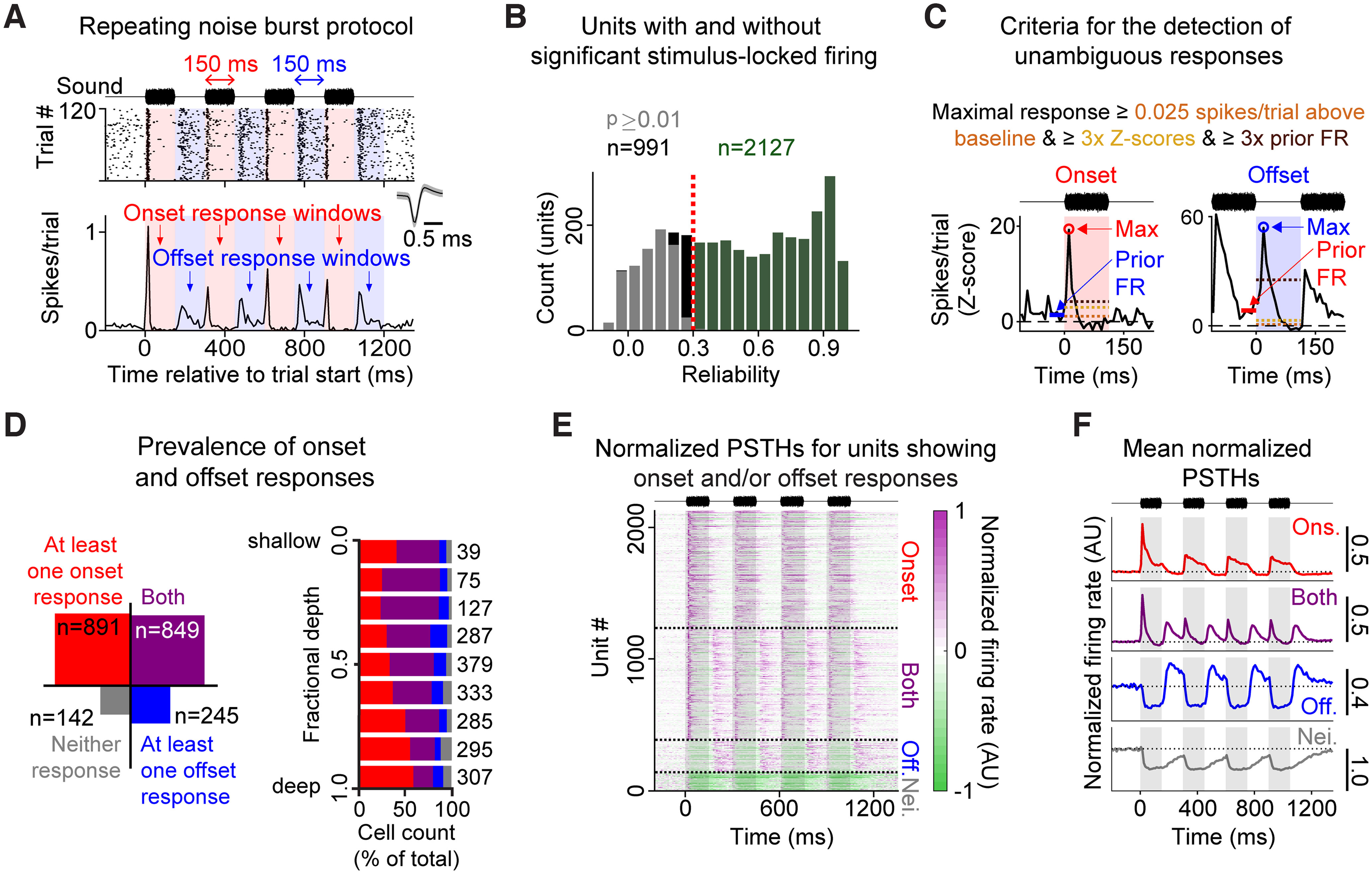
Identifying onset- and offset-responsive cells. ***A***, Spike raster (top) and PSTH (bottom) showing firing from an example unit. Red shading represents onset response windows. Blue shading represents offset response windows. This unit responded both to the sound (onset) and the termination of the sound (offset). ***B***, Histogram represents the spike-time reliability scores for all single units. Of 3118 single units, 2127 (green) showed a reliability score that was ≥0.3 and statistically significant (*p* < 0.01) (other units were not included in further analyses). ***C***, Criteria for identifying unambiguous onset and offset responses. The PSTHs show example onset (left) and offset (right) responses. The response maxima (circles) exceed the 0.025 spikes/bin/trial above baseline threshold (orange dashed line), the 3× *z*-score threshold (dark yellow dashed line), and the 3× prior FR threshold (dark brown dashed line). ***D***, Left, Count of units showing ≥1 onset response (red), ≥1 offset response (blue), both onset and offset responses (purple), or neither response type (gray). Right, Count of onset- and/or offset-responsive units as a function of cortical depth. ***E***, Normalized PSTHs, grouped according to whether they showed onset responses only, onset and offset responses, offset responses only, or neither onset nor offset responses. ***F***, Mean normalized PSTHs for all units. Groups are the same as in ***D*** and ***E***.

To measure frequency tuning, we used 14 tones of different frequencies, logarithmically spaced between 4 and 64 kHz. Each tone was 100 ms long, ramped on and off using 5 ms linear ramps. These tones were separated by silent periods of 300 ms, randomly interleaved, and repeated 70 times. To measure history dependence, we used a stimulus consisting of 4 × 150 ms noise bursts (Fig. 1*A*) separated (internoise interval) by 150 ms of silence. Each noise burst consisted of white noise bandpass filtered between 4 and 64 kHz, and was ramped on and off using 5 ms linear ramps. This stimulus was presented 120 times with an intertrial interval of 8 s.

### Data analysis

#### Response peristimulus time histograms (PSTHs)

**Figure 2.**
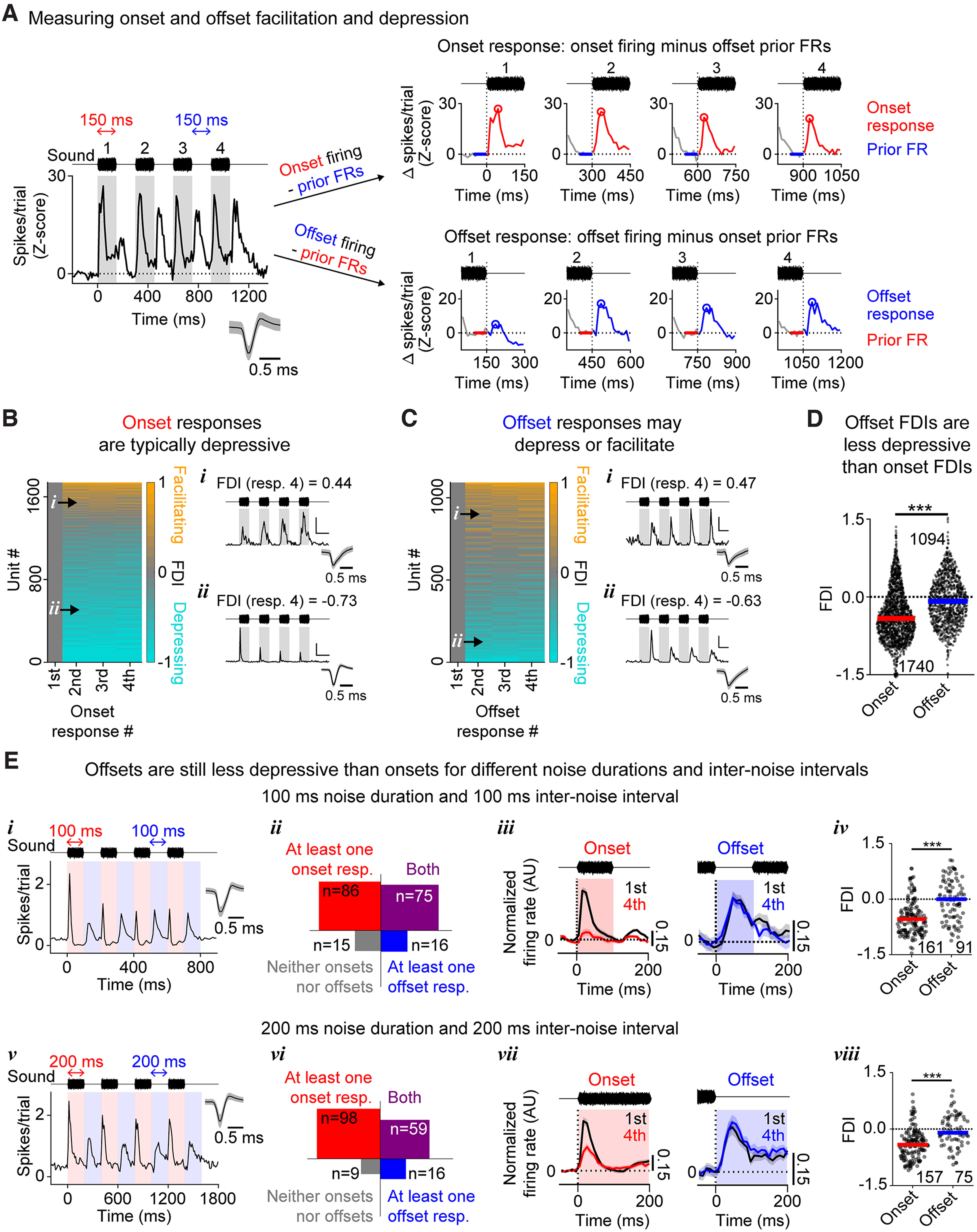
Offset responses are less depressive than onset responses. ***A***, Before calculating the maximal FR in a response window (***A***, right, circles), the FR at the end of the prior response window (***A***, right, horizontal bars) was subtracted. ***B***, Left, Onset FDIs for all units showing at least one onset response. PSTHs (***Bi***,***Bii***) show firing from example units with facilitatory (***Bi***) or depressive (***Bii***) responses. ***C***, Same as in ***B***, but for offset responses. ***D***, Scatter-violin plot showing the FDI from the fourth onset (red) and offset (blue) response. Offset FDIs were significantly more positive than onset FDIs (*p* < 0.001). ***E***, Onset and offset history dependence of responses to a stimulus consisting of either four 100 ms noise bursts separated by silent intervals of 100 ms (***Ei–Eiv***) or four 200 ms noise bursts separated by silent intervals of 200 ms (***Ev–Eviii***). ***Ei***, ***Ev***, Firing from example units in response to these stimuli. ***Eii***, ***Evi***, Count of units showing onset and/or offset firing in response to these stimuli. ***Eiii***, ***Evii***, Mean normalized PSTHs for the first (black) and fourth (red) onset response (left), or the first (black) and fourth (blue) offset response (right). All units showed either ≥1 onset response (left) or ≥1 offset response (right). Shaded error bars indicate SEM. ***Eiv***, ***Eviii***, Onset and offset FDIs for all units depicted in ***Eiii*** (***Eiv***) or ***Evii*** (***Eviii***). Offset FDIs were significantly more positive than onset FDIs for both stimuli (***Eiv***,***Eviii***, *p* < 0.001). ***p < 0.001.

**Figure 3.**
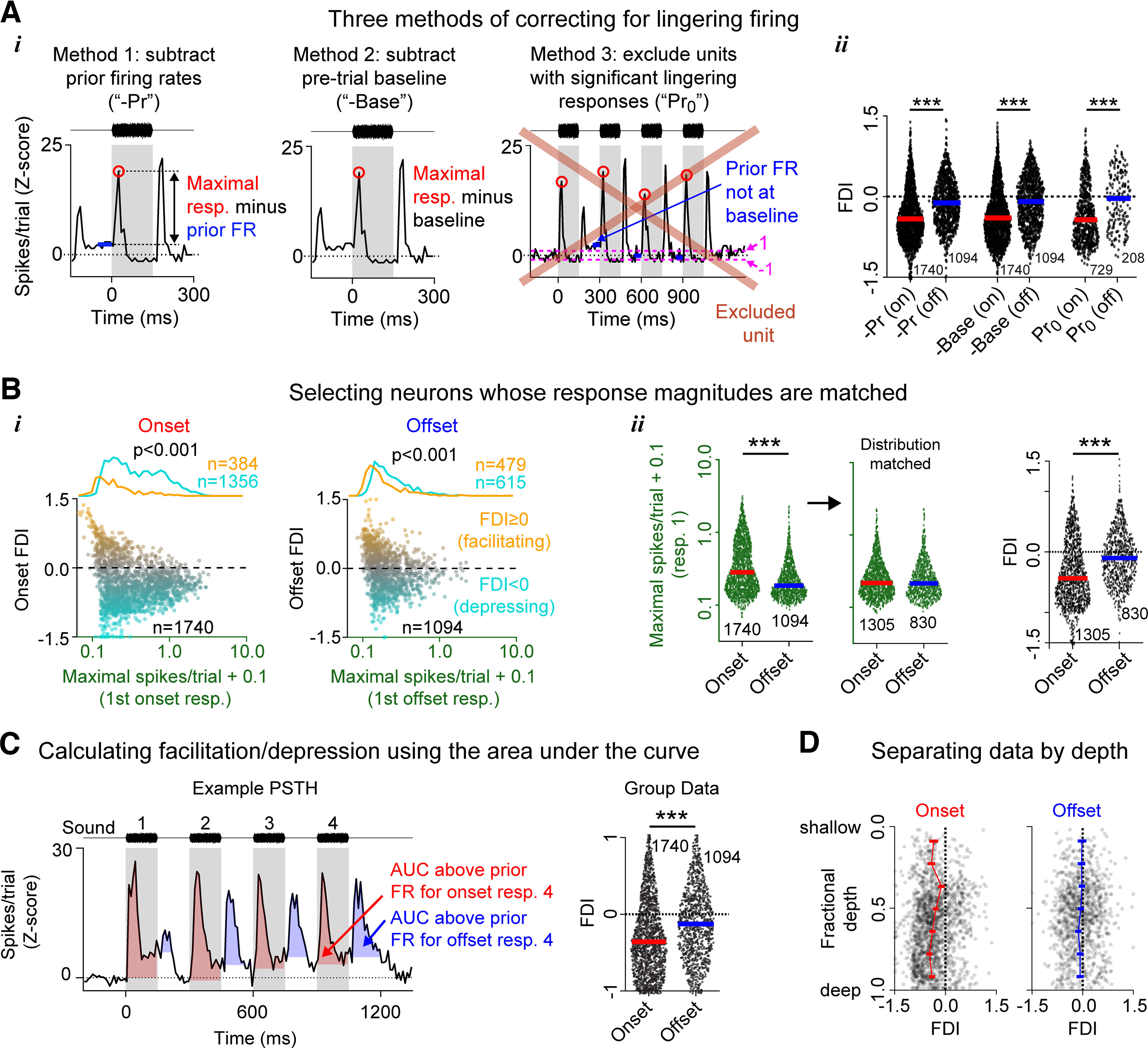
Offsets remain less depressive than onsets, even when different analysis choices are made. ***Ai***, To assess the effect of prior FR subtraction on measured FDIs, we compared FDIs calculated in three different ways: using response maxima from which the immediately preceding FRs were subtracted (left, Method 1: -*Pr*), using response maxima from which the baseline firing was subtracted (middle, Method 2: *-Base*), and using baseline subtraction, but only including cells for which all four prior FRs returned to within 1 SD of baseline (right, Method 3: *Pr_0_*). ***Aii***, Onset and offset FDIs for the three prior FR conditions (*-Pr*, *-Base*, *Pr_0_*). A two-way ANOVA revealed a significant effect of response type (onset and offset, *p* < 0.001), but not calculation method (*p* = 0.95). ***Bi***, The FDI was plotted against the response maximum (in spikes/trial) for Response 1, for onset (left) and offset (right) responses. Marginal histograms (top) represent the distributions of first response maxima for cells with negative (cyan, depressive) or positive (orange, facilitative) FDIs. For both onset and offset responses, cells with negative/depressing FDIs on average had stronger first responses than cells with positive/facilitating FDIs (onset: *p* < 0.001; offset: *p* < 0.001). ***Bii***, Across all cells, onset responses typically showed higher maximal FRs than offset responses (left, *p* < 0.001). Among cells matched for maximal FRs (middle), the offset FDIs were still significantly more positive than the onset FDIs (right, *p* < 0.001). ***Bi***, ***Bii***, 0.1 was added to all spikes/trial values to allow plotting these data on a log scale (for strongly facilitating or depressing cells, the maximal baseline-subtracted response within a given window occasionally fell between −0.1 and 0). ***C***, PSTH (left) from an example unit showing the onset (red shading) and offset (blue shading) AUCs. AUCs are calculated with respect to the mean FR just before the response window (i.e., prior FRs). Right, Offset AUC FDIs were significantly more positive than onset AUC FDIs (*p* < 0.001). ***D***, Onset (left) and offset (right) FDI as a function of cortical depth. Horizontal error bars indicate SEM. ***p < 0.001.

**Figure 4.**
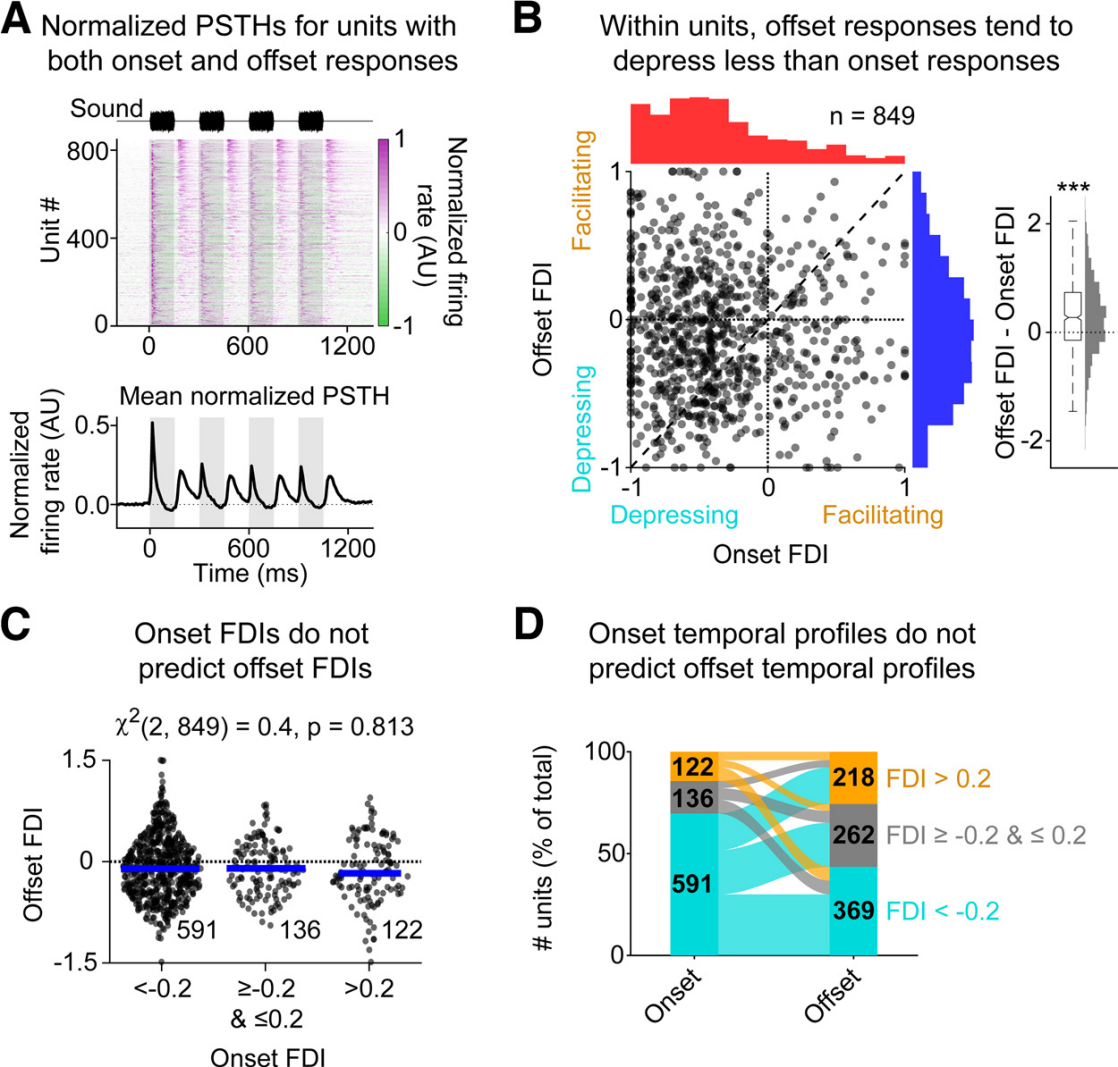
Within cells, onset history dependence does not correlate with offset history dependence. ***A***, Normalized PSTHs (top) and the mean of these normalized PSTHs (bottom) from all units showing both onset and offset responses (*n* = 849). Rows are organized by the maximum offset response amplitude for each unit. ***B***, The offset FDI plotted against the onset FDI, for all units showing both onset and offset responses (left). Right, The difference between offset and onset FDIs shows that offset FDIs tend to be significantly more positive (more facilitating) than onset FDIs in the same units (*p* < 0.001). ***C***, The offset FDI for units that had both onset and offset responses, split according to whether the onset FDI was very negative (<−0.2, left), around zero (≥−0.2 and ≤0.2, middle), or very positive (>0.2, right). No significant difference was found between groups. ***D***, The alluvial plot represents the diverse relationships between onset and offset temporal response profiles within units. Cyan represents depression, FDI <−0.2. Gray represents stable, FDI ≥−0.2 and ≤0.2. Orange represents facilitatory, FDI >0.2. Numbers on bars indicate number of cells. ***p < 0.001.

To measure responses to the 4× noise burst stimulus, we calculated PSTHs from 200 ms before the start of the first noise burst to 150 ms following the end of the last noise burst at a 10 ms time resolution, and *z*-scored them using the mean and SD of the baseline (calculated based on the 4 s period of spontaneous activity before the start of the stimulus on all 120 trials). These *z*-scored PSTHs were the bases for analyses in Figures 1–4 and Figures 6–8. For visualization and for the analyses in Figure 9, these PSTHs were further normalized by dividing the PSTH by its absolute maximum (in accordance with Seay et al., 2020).

#### Stimulus-locked firing (reliability)

To determine whether a cell's firing was significantly related to the history dependence stimulus, we calculated PSTH reliability as follows. For each cell, we calculated the cross-correlation coefficient between one PSTH, generated from a random half of all stimulus presentations, and a second PSTH generated from the other half. This process was repeated 1000 times, and we defined “PSTH reliability” to be the mean cross-correlation coefficient of all 1000 iterations. To determine statistical significance, we obtained a null distribution of mean reliability scores from PSTHs generated from shuffled spike trains (shuffled interspike intervals). *p* values were adjusted using the Benjamini–Hochberg procedure for false discovery rate correction (Benjamini and Hochberg, 1995). A neuron with stimulus-locked firing was defined as a neuron with a significant (*p* < 0.01) reliability score ≥0.3 (see Fig. 1*B*).

#### Definition of unambiguous onset and offset responses

For each of the four noise bursts in the stimulus, we defined the onset response window as the 150 ms during the noise burst (see Fig. 1*A*, red shading) and the offset response window as the 150 ms of silence following the noise burst (see Fig. 1*A*, blue shading). We identified a response window as containing an unambiguous response if the maximum firing rate (FR) in the window exceeded each of three thresholds (see Fig. 1*C*): (1) 0.025 spikes/bin/trial above baseline, (2) 3× *z* scores above baseline, and (3) 3× the mean FR during the last 50 ms of the prior response window. An onset response window in which the maximal FR exceeded all three thresholds was considered an onset response, and a cell showing an onset response to at least one of the four noise bursts was considered onset-responsive. Similarly, a sound-offset response window in which the maximal FR exceeded all three thresholds was considered an offset response, and a cell showing an offset response to at least one of the four noise bursts was considered offset-responsive.

#### Quantification of history dependence

To quantify history dependence, we calculated a facilitation-depression index (FDI), as follows: the FDI for the n^th^ onset response was defined as the difference between the magnitudes of the n^th^ response and the first response, divided by the maximum of all four responses. FDIs for offset responses were calculated by applying this same algorithm to the offset response measurements. If the n^th^ response is less than the first response (i.e., if it is depressed), this value will be <0. If the n^th^ response is facilitated with respect to the first response, this value will be >0. Because the strength of facilitation or depression was typically consistent from response to response and strongest for Response 4, in all cases outside of Figure 2*B*, *C*, “FDI” refers to the FDI for Response 4.

#### Hierarchical clustering of PSTH spike trains

To group cells according to spiking responses, we calculated the PSTH responses to our 4× noise burst protocol at a 10 ms time resolution and normalized them to have a baseline of 0 and a maximum absolute value of 1. We performed hierarchical clustering on these PSTHs using the time period from the start of the first noise burst to 150 ms following the end of the last noise burst. To perform clustering, we first used the 'linkage' function in MATLAB to create linkage trees, using a correlation-based distance metric. We then used the MATLAB function 'cluster' to split these linkage trees into a predefined number of clusters. Clusters with <10 units were dropped.

#### Classification of different cell types using the action potential waveform and opto-tagging

**Figure 5.**
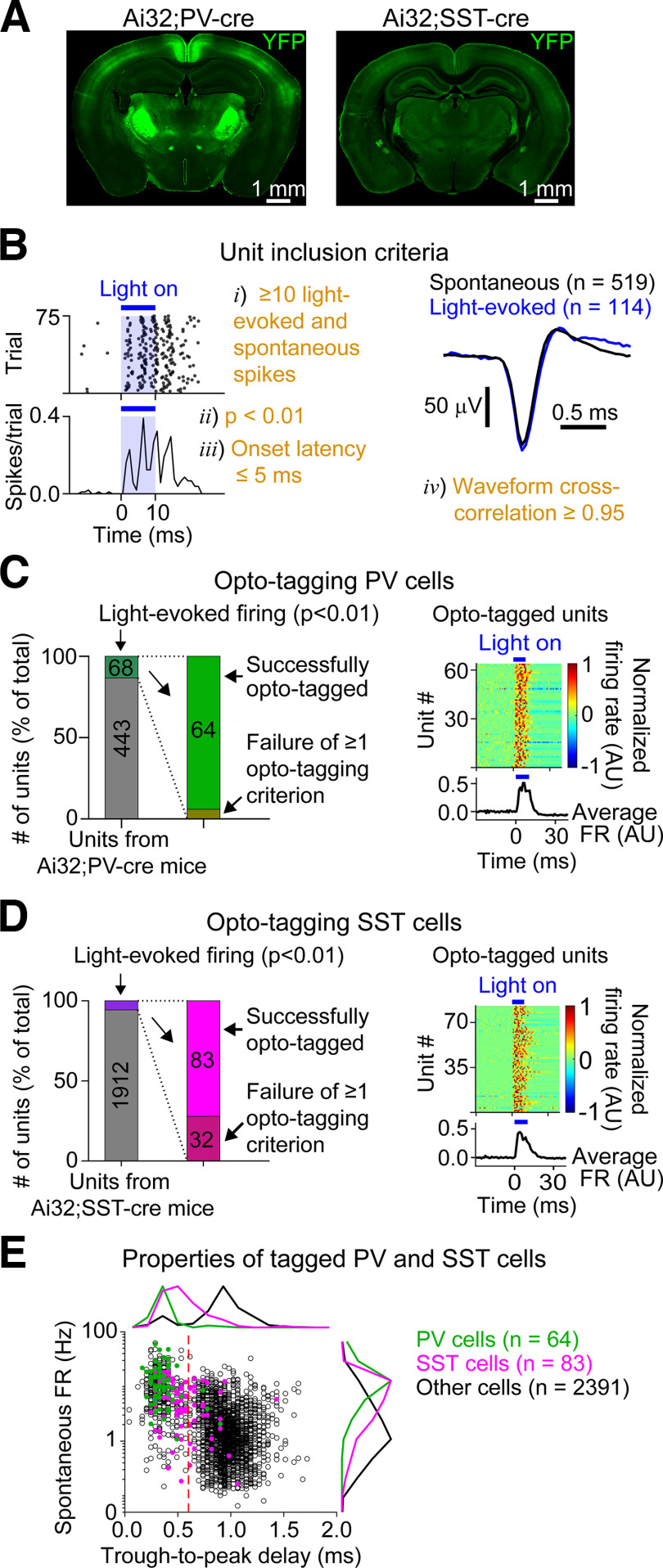
Opto-tagging PV and SST neurons. ***A***, Histology showing eYFP fluorescence (green) from a representative PV-Cre;Ai32 (left) and SST-Cre;Ai32 (right) mouse using coronal slices (50 μm thickness) containing the AC. ***B***, Identifying opto-tagged cells. The photo-tagging stimulus consisted of 75 trials of a 10 ms blue light pulse, each interspersed by a 1.5 s intertrial interval (left). The waveforms on the right show the mean spontaneous (black) and light-evoked (blue) waveform for the same cell depicted on the left (cross-correlation between the two waveforms = 0.99). Orange text (labeled i-iv) represents the criteria required for a cell to be classified as successfully opto-tagged. ***C***, Opto-tagged PV cells. From the 511 units recorded from all PV-Cre;Ai32 mice used in this study, 68 showed significantly elevated light-evoked firing, and 64 of these units passed all remaining opto-tagging criteria (left). Normalized PSTHs on the right (right, top) and the mean of these normalized PSTHs (right, bottom) show the light pulse responses of these 64 successfully opto-tagged PV cells. Each row in the top panel is a normalized PSTH from a single unit (amplitude expressed using color scale). ***D***, Opto-tagged SST cells. Format the same as in ***C***. Of the 2027 units recorded from all SST-Cre;Ai32 mice used in this study, 115 cells showed significantly elevated light-evoked firing, and 83 of these units passed all remaining opto-tagging criteria. ***E***, Spontaneous FR plotted against the waveform trough-to-peak delay for all units recorded from PV-Cre;Ai32 (green) and SST-Cre;Ai32 (magenta) mice. The marginal histograms are normalized to have a maximum of 1. Nontagged (“Other”) cells were typically broad-spiking (trough-to-peak delay ≥600 μs, red dashed line). PV and SST cells were typically narrower spiking and showed higher spontaneous FRs.

We classified cells according to their action potential waveform trough-to-peak delay time. In rodents, nearly all cells with narrow spiking (NS) action potentials are inhibitory interneurons, while most, but not all, cells with broad spiking (BS) action potentials are excitatory, pyramidal neurons (Barthó et al., 2004). Consistent with previous reports in mice (Stark et al., 2013; Rummell et al., 2016; Bigelow et al., 2019), the distribution of trough-to-peak delay times in our dataset was bimodal (see Fig. 5*E*). The dip between peaks in the bimodal distribution occurred at 600 μs (see Fig. 5*E*, vertical red dashed line), and this value served as our boundary to split cells into NS and BS cells. In accordance with previous reports (e.g., Niell and Stryker, 2008; Bigelow et al., 2019; Ryu et al., 2019), the spontaneous FR of NS cells was typically faster than BS cells (10.2 ± 8.8 Hz vs 2.3 ± 2.9 Hz, Mann–Whitney *U* test: *p* < 0.001).

**Figure 6.**
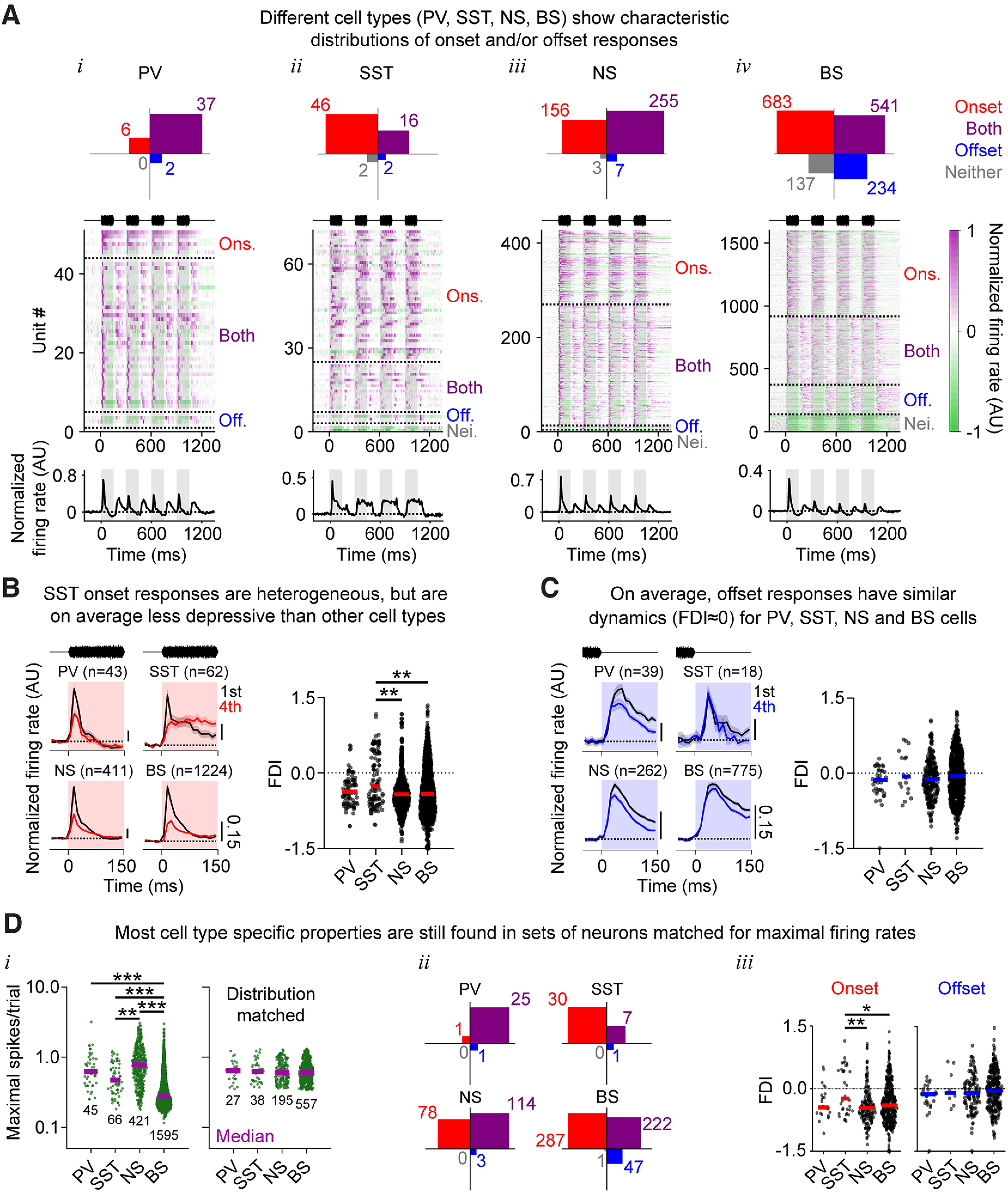
Unlike onset history dependence, offset history dependence is similar across cell types. ***A***, Top row, Distribution of units showing onset responses only (red), offset responses only (blue), both response types (purple), or neither response type (gray), for PV (***Ai***), SST (***Aii***), NS (***Aiii***), and BS (***Aiv***) cells. Middle and bottom rows, Normalized PSTHs (middle) and the mean of these normalized PSTHs (bottom), split according to cell type. ***B***, Facilitation and depression of onset responses in different cell types. Left, Mean normalized PSTHs for the first and fourth onset responses (black and red, respectively), for each of the four cell populations. Right, The onset FDIs for each cell population show that onset FDIs were significantly more positive for SST cells than NS (*p* < 0.01) and BS (*p* < 0.01) cells. ***C***, Same as in ***B***, but for offset responses. Offset FDIs did not differ significantly between cell types (Kruskal–Wallis test: *p* = 0.28). ***Di***, Maximal FRs (maximal PSTH spikes/trial values) from the start of the first noise burst to 150 ms past the last noise burst, split according to cell type (PV, SST, NS, BS). Cell types had significantly different FRs (*p* < 0.001, Dunn's *post hoc* tests). Right, Distribution matching was performed to select a subpopulation of cells with similar FRs. ***Dii***, For all cells, distribution matched for FR (***Di***, right), the proportion of units showing onset responses only (red, top left), offset responses only (blue, bottom right), both response types (purple, top right), or neither response type (gray, bottom left), remained similar to the data in the population overall (***A***, top row). ***Diii***, Similarly, following the distribution matching procedure, cell type differences in onset and offset FDIs remained similar to the entire population (***B***,***C***). *p < 0.05; **p < 0.01; ***p < 0.001; Dunn's *post hoc* tests.

**Figure 7.**
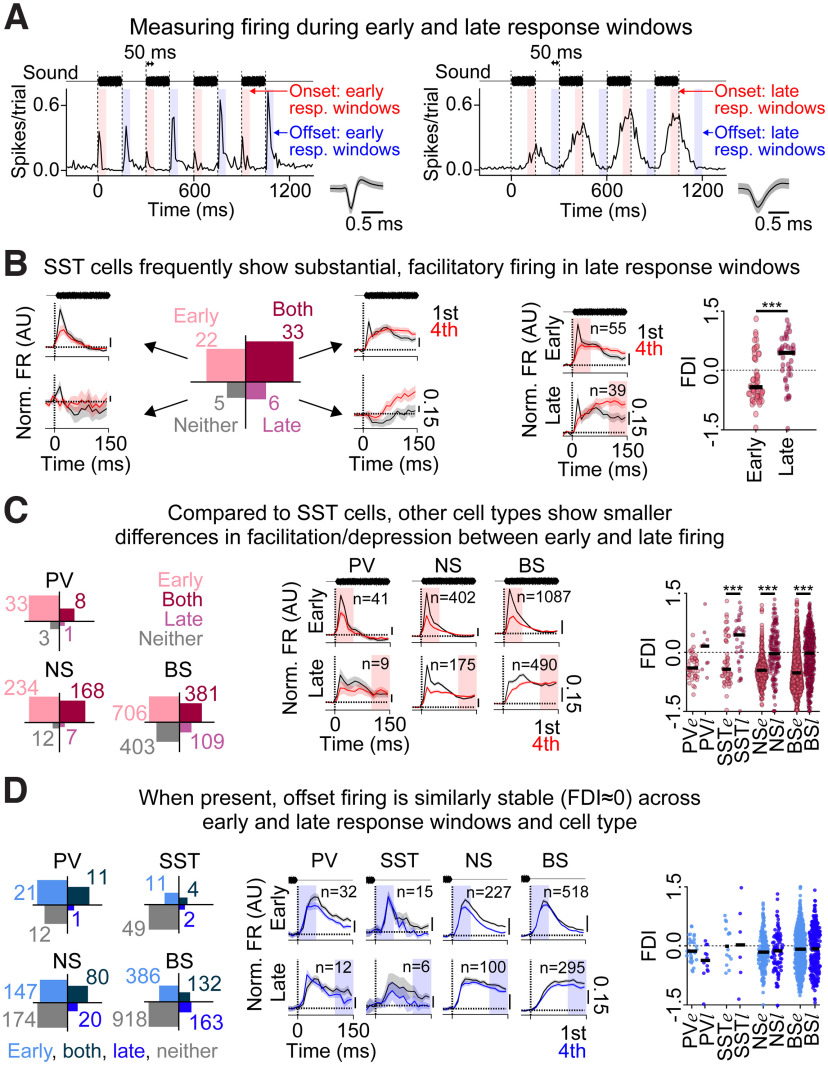
Onset but not offset history dependence differs for early versus late firing. ***A***, PSTHs from example units showing early (left) and late (right) response windows, for onset (red shading) and offset (blue shading) responses. Early and late onset and offset responses were detected using the thresholding technique depicted in Figure 1*C*. ***B***, Left, Count of SST cells showing responses in the early onset response windows only (top left, light pink), the late onset response windows only (bottom right, dark pink), the early and late onset response windows (top right, maroon), or neither the early nor late onset response windows (bottom left, gray). The surrounding plots represent the mean normalized PSTHs for these four subpopulations, for the first (black) and fourth (red) onset responses. ***B***, Right, For SST cells, the FDI was significantly more positive for late onset firing than early onset firing (*p* < 0.001). ***C***, Similar to ***B***, but including PV, NS, and BS cells. Left, Count of cells showing onset responses in early and/or late response windows. Middle, Mean normalized PSTHs for the first (black) and fourth (red) onset response, for early (top) and late (bottom) response windows. Right, The FDI for early and late onset response windows. ***D***, Same as in ***C***, but showing results for offset responses for PV, SST, NS, and BS cells. ***p < 0.001 (*post hoc* Tukey tests).

In some experiments, we also identified PV^+^ or SST^+^ cells by photostimulating ChR2 (see Fig. 5). We activated these cells using a 10 ms blue (470 nm) light pulse (75 trials, 1.5 s intertrial interval). The blue light was delivered using a fiber-coupled LED light source (Mightex), and the light intensity was adjusted individually for each recording until light-activated firing in the absence of opto-artifact was observed. The following criteria had to be met for a cell to be classified as directly activated by the light pulse (i.e., 'opto-tagged'): (1) at least 10 light-evoked and 10 spontaneous action potentials during the opto-tagging protocol, (2) significantly increased light-evoked firing with respect to baseline (*p* < 0.01, one-sided Wilcoxon signed-rank test), (3) light-evoked firing with an onset latency ≤5 ms (defined below), and (4) a cross-correlation coefficient of ≥0.95 between the mean light-evoked and mean spontaneous waveforms (i.e., the voltage signals between −330 and 570 μs from the time of the action potential trough). The minimum of 10 spikes (Criterion 1) ensured that enough waveforms were available to generate the means required for the correlation analysis (Criterion 4). In Figures 6, 7, and 9, neurons showing significantly elevated light-evoked firing but failing on one or more of the other opto-tagging criteria were not included in the BS and NS groups.

#### Measuring tone- or light-evoked onset latency

We generated PSTHs that had 2 ms time bins and smoothed them using a Savitsky–Golay filter (third-order, 10 ms window). The onset latency was defined as the first time bin in which evoked firing exceeded 2.5 SDs above baseline (baseline: 100 ms period of spontaneous activity before the start of the stimulus).

#### Identifying recording sites with primary-like onset latencies and clear signs of frequency tuning

Previous research has shown that neurons in primary cortical fields typically fire with shorter onset latencies than neurons in secondary cortical fields (e.g., 5-18 ms vs 8-32 ms) (Joachimsthaler et al., 2014), and show stronger frequency tuning (Morrill and Hasenstaub, 2018). Therefore, in this study, we only analyzed recordings from sites with relatively quick onset latencies and clear frequency tuning. We measured multiunit firing in response to pure tones (collapsed across frequency) and designated a recording as showing primary-like onset latencies if the median onset latency from all channels showing significantly elevated tone-evoked firing (*p* < 0.01) was ≤14 ms. We calculated multiunit frequency tuning curves (FTCs) in response to our tone stimuli and classified each channel as showing frequency tuning if the following criteria were met: (1) the tone-evoked FR at best frequency exceeded 50% of the mean tone-evoked FR for all non-best frequency tones, and (2) the FTC variance (variance of the mean FRs across frequency) exceeded that expected by chance at *p* < 0.01, determined by creating a null distribution of FTC variance values (1000 iterations) using shuffled spike trains (shuffled interspike intervals). We included recordings in our analysis if ≥50% of the channels with significantly elevated tone-evoked firing passed these two tuning criteria and if it had primary-like onset latencies.

#### Cortical depth estimation

We used recording probes that are longer (1417 μm) than the span of the mouse cortex (∼800 μm). This allowed us to record above (pia) and below (white matter) the cortex, and thus estimate cortical depth using the following procedure: we presented awake mice with clicks (5 ms nonramped white noise bursts) and tones (see Sound stimuli), created click- and tone-evoked PSTHs, and subsequently used these to visually determine the shallowest and deepest channels with sound-evoked activity. For all channels within these boundaries, we assigned a normalized depth measurement to each channel (fractional depth), where the deepest channel has a value of 1 and the shallowest channel has a value of 0. These methods have previously been described in detail in Bigelow et al. (2022).

#### Distribution matching for FRs

For some analyses, we wanted to compare the degree of facilitation/depression across groups of cells with similar FRs (see, e.g., Figs. 3*B*, 6*D*). To do this, we performed the following distribution matching procedure to match FRs across groups: first, we binned the FRs for each group into 6 bins (e.g., for each cell type, or cluster), and normalized these bins by the total number of cells in the group (i.e., converted to probability distributions). Second, we found the area of overlap of these probability distributions (i.e., for each bin in the distribution, the minimum of the probability distributions for that bin across all groups); we call this distribution the overlap distribution. Last, the percentages in this overlap distribution were used to randomly select cells to keep for further analysis. For example, say you have 10 cells in bin *y* (e.g., 10-20 Hz) for group *x*, and these 10 cells make up 10% of the total for this group (*n* = 100), but the overlap distribution for this bin is only 5%. In this case, for this group and bin, 5 of the 10 cells will be randomly selected and taken for further analysis.

#### Finding cell populations with matched onset and offset response dynamics

To find populations of onset-responsive and offset-responsive cells for which the latency and duration of the first response were similar, we first found all cells whose responses to the first sound offset were transient (i.e., where the maximum response occurred within the first 50 ms, and for which the mean FR during the last 50 ms of the response had a *z* score <1). We then used the average of these transient offset responses as a template. As our dynamics-matched onset-responsive group, we selected all cells whose initial onset responses matched this template with a correlation coefficient (*r*^2^) of ≥0.75. Similarly, for our dynamics-matched offset-responsive group, we selected all cells whose initial offset responses matched this template with a *r*^2^ of ≥0.75.

### Histology

To determine whether PV-Cre and SST-Cre were correctly expressed in our Pv-Cre;Ai32 and SST-Cre;Ai32 mouse lines, following the final electrophysiological recording from each mouse, the mouse was killed and the brain was placed into PFA (4%) for ∼24 h and then sucrose (30%) for at least 2 d. We cut 50-µm-thick coronal brain slices containing the whole forebrain using a microtome (Microm HM 450). The slices were mounted onto slides, and eYFP fluorescence was captured using a fluorescence microscope (Keyence BZ-X810). eYFP was excited at a wavelength of 470 ± 40 nm, and the emission was collected at 525 ± 50 nm. Two of 5 PV-Cre;Ai32 brains and 0 of 8 SST-Cre;Ai32 brains showed at least some misexpression of the Cre-dependent reporter (compared with our laboratory's reference atlases of fluorescence in these crosses), and were excluded from opto-tagging analyses.

### Statistics

Statistical analysis was performed using MATLAB. When testing for a difference between two or more groups, we first tested whether all groups showed a normal distribution (*p* > 0.05 using a Shapiro–Wilk test), and had homogeneous variance (*p* > 0.05 using a Levene's test). These assumptions were not met for any of our group comparisons, and thus we only used nonparametric tests. To compare two unpaired data groups (see, e.g., Fig. 2*D*), we used Mann–Whitney *U* tests. To compare two paired data groups (see, e.g., Fig. 4*B*, right), we used Wilcoxon-signed rank tests. To compare three or more unpaired data groups (see, e.g., Fig. 4*C*), we used Kruskal–Wallis tests. Following a significant result from a Kruskal–Wallis test, Dunn–Sidak *post hoc* comparison tests were used to test for differences between individual groups. To compare the effect of two independent variables (see, e.g., Fig. 7*C*,*D*, right), we performed two-way ANOVAs with *post hoc* Tukey tests. To account for our data being nonparametric, these two-way ANOVAs were performed on ranked data using the aligned rank transform toolbox in R (Wobbrock et al., 2011). For all plots showing FRs or FDIs (see, e.g., Fig. 2*D*), the horizontal bars represent the median. Error bars always indicate the SEM.

## Results

We recorded action potentials from 64 channel linear silicon probes implanted in the primary AC of *n* = 18 awake mice (male and female). We presented 120 trials of a sound stimulus consisting of four noise bursts, each 150 ms in duration and separated by 150 ms of silence. Each stimulus was followed by 8 s of silence (intertrial interval). This stimulus allowed us to measure changes in sound-evoked (“onset”) and sound-offset (“offset”) response magnitudes across repetitions of the same stimulus. Firing from an example unit is shown in Figure 1*A*.

### Onset and offset firing in AC

Among the 3118 stable, well-isolated single units recorded from primary AC, 2127 (∼68%) showed significant stimulus-locked firing to this protocol (Fig. 1*B*, reliability ≥0.3 and significant at *p* < 0.01; see Materials and Methods). Only these units were analyzed further. We constructed PSTHs using 10 ms bins, defined onset and offset response windows for each of the four noise bursts (Fig. 1*A*), and defined a window as containing an unambiguous response if the maximal FR within the window exceeded 0.025 spikes/bin/trial, had a *z* score >3, and was at least 3× the mean FR in the 50 ms before the window (Fig. 1*C*). The 0.025 spikes/bin/trial and 3× *z*-score thresholds ensured that stimulus-driven firing exceeded spontaneous firing and was large enough to not be swamped by noise. The 3× prior FR threshold ensured that stimulus-driven firing unambiguously exceeded any lingering firing from a prior response. A unit that had onset responses to at least one of the four noise bursts was considered onset-responsive, and a unit that had offset responses to at least one of the four noise bursts was considered offset-responsive. Among the 2127 units with significant stimulus-locked firing (Fig. 1*B*), 891 (∼42%) showed onset responses only, 245 (∼12%) showed offset responses only, 849 (∼40%) showed both onset and offset responses, and 142 (∼7%) showed neither onset nor offset responses (these neurons were typically inhibited by the test stimulus) (Fig. 1*D–F*). These results differed depending on cortical depth (Fig. 1*D*, right). Cells in the shallower layers were more likely to show offset responses, whereas cells in the deeper layers were more likely to show onset responses only. These results are consistent with previous studies, which show that a substantial proportion of onset-responsive units also respond to sound offsets (>25% in previous studies; roughly half in this study), and onset firing is more common than offset firing (Sołyga and Barkat, 2019, 2021; Chong et al., 2020; Li et al., 2021).

### Quantifying history dependence

We first aimed to quantify the prevalence of response facilitation or depression among onset and offset responses. For this, we had to make two analysis choices: first, how to measure response magnitude (e.g., as maximal response height, area under the curve [AUC], etc). Second, because in this dataset many cells showed responses that were so sustained as to bleed into the next response window (e.g., the first onset response in Fig. 2*A*, left), we had to choose whether to measure responses with respect to the pretrial baseline or with respect to the FR at the end of the prior response. We first analyzed facilitation and depression using the difference between the maximal response and the prior FR (Fig. 2*A*). In order to compare results across cells with very different FRs, we normalized changes in response magnitude to produce an FDI: for each onset response, we divided the change in response magnitude with respect to the first onset response by the size of the largest onset response, and we calculated offset response FDIs analogously but using only offset response magnitudes. If a response is facilitated with respect to the first response, then its FDI will be >0; and if a response is depressed with respect to the first response, its FDI will be <0.

### Offset responses tend to be less depressive than onset responses

Figure 2*B*, *C* shows onset and offset FDIs from all units exhibiting at least one onset (Fig. 2*B*) or offset (Fig. 2*C*) response. Onset responses were more likely to have negative FDIs (i.e., more likely to depress than facilitate), whereas offset responses were roughly equally likely to have positive or negative FDIs (i.e., to be facilitating or to be depressing). Because the direction of response change was typically uniform across Responses 2-4, and depression or facilitation was typically strongest for Response 4, we performed all subsequent analyses on FDIs from Response 4 only. Indeed, the FDIs for offset Response 4 were significantly more positive than the FDIs for onset Response 4 (Fig. 2*D*, −0.35 ± 0.50 vs −0.07 ± 0.45, Mann–Whitney *U* test: *p* < 0.001), implying that for these stimuli, offset responses were less depressive than onset responses. Because previous studies have shown that the strength of depression and facilitation varies depending on interstimulus intervals (Brosch and Schreiner, 1997; Wehr and Zador, 2005), we wondered whether this result was specific to these particular stimulus timings. To test this, we recorded 15 penetrations from an additional 5 male and female mice, in which we presented randomly interleaved trials of repeating noise burst stimuli with different temporal characteristics (either 100 ms noise bursts with 100 ms internoise intervals, or 200 ms noise bursts with 200 ms internoise intervals; 240 trials in total) (Figs. 2*Ei*,*Ev*). Changing stimulus timing did not substantially alter the proportion of cells showing onset and/or offset responses (Figs. 2*Eii*,*Evi*, for 100 ms noise bursts and 100 ms internoise intervals, for 192 cells in total: onset only: ∼44%, onset and offset: ∼39%, offset only: ∼8%, neither: ∼8%; for 200 ms noise bursts and 200 ms internoise intervals, for 182 cells in total: onset only: ∼54%, onset and offset: ∼32%, offset only: ∼9%, neither: ∼5%), and offsets consistently remained less depressive than onsets (Fig. 2*Eiii*,*Eiv* and Fig. 2*Evii*,*Eviii*, for 100 ms noise bursts and 100 ms internoise intervals: onset FDI: −0.51 ± 0.45, offset FDI: −0.01 ± 0.56, Mann–Whitney *U* test, *p* < 0.001; for 200 ms noise bursts and 200 ms internoise intervals: onset FDI: −0.40 ± 0.40, offset FDI: −0.04 ± 0.43, Mann–Whitney *U* test, *p* < 0.001). These results suggest that offset responses are more facilitative than onset responses, regardless of stimulus timing.

### Offset responses are still less depressive than onset responses when different analysis choices are made

We wondered whether our results were confounded by lingering firing driven by the prior stimulus in the noise burst (“prior FRs”), as this firing frequently bled across response windows (e.g., Fig. 2*A*, left). To test for this, we compared onset and offset FDIs calculated in three different ways. First, we calculated FDIs using prior FR-subtracted responses, as in the previous analysis (Fig. 3*Ai*, left; data identical to Fig. 2). Second, we calculated FDIs for the same cells, but using baseline-subtracted responses (Fig. 3*Ai*, middle). Third, we calculated FDIs using baseline-subtracted responses, but only from cells in which all four prior FRs returned to within 1 SD of baseline (Fig. 3*Ai*, right). We used a two-way ANOVA to analyze the effect of the calculation method on onset and offset FDIs (Fig. 3*Aii*). This test revealed a nonsignificant interaction between response type (onset vs offset) and calculation method (*F*_(2, 6602)_ = 0.05, *p* = 0.95), and simple main effects analyses revealed that the FDI differed significantly between onsets and offsets (*F*_(1, 6603)_ = 341.4, *p* < 0.001), but not between the three calculation methods (*F*_(2, 6602)_ = 0.49, *p* = 0.95). *Post hoc* Tukey tests showed that offsets were significantly less depressive than onsets for all three calculation methods (Method 1: onset FDI: −0.36 ± 0.50, offset FDI: −0.07 ± 0.45, *p* < 0.001; Method 2: onset FDI: −0.34 ± 0.49, offset FDI: −0.06 ± 0.47, *p* < 0.001; Method 3: onset FDI: −0.34 ± 0.51, offset FDI: −0.05 ± 0.51, *p* < 0.001). These results show that offsets are more facilitatory than onsets, regardless of how prior FRs are handled.

We additionally wondered whether our results related to differences in the magnitude of firing evoked by sound onsets and sound offsets. One mechanistic explanation for response depression is that the underlying synapses may be undergoing short-term synaptic depression through vesicle depletion, which is more prominent at stronger synapses with higher release probabilities (Tsodyks and Markram, 1997; Schneggenburger et al., 2002). Because of this, cells with stronger initial responses may show stronger depression. Indeed, in our dataset, cells with larger initial responses (i.e., in response to the first noise burst) were generally more depressive than cells with smaller initial responses, and this was true for both onset responses (Fig. 3*Bi*, left, 0.42 ± 0.46 spikes/trial vs 0.16 ± 0.25 spikes/trial, Mann–Whitney *U* test, *p* < 0.001) and offset responses (Fig. 3*Bi*, right, 0.19 ± 0.24 spikes/trial vs 0.12 ± 0.20 spikes/trial, Mann–Whitney *U* test: *p* < 0.001). Moreover, onset responses were stronger than offset responses (Fig. 3*Bii*, left, 0.37 ± 0.44 spikes/trial vs 0.32 ± 0.46 spikes/trial, Mann–Whitney *U* test, *p* < 0.001), which suggests that they may be more liable to depress. However, even when comparing populations of cells with equivalent initial (first) response sizes (Fig. 3*Bii*, middle), offsets were still significantly less depressive than onsets (Fig. 3*Bii*, right, mean FDI: −0.32 ± 0.58 vs −0.08 ± 0.45, Mann–Whitney *U* test: *p* < 0.001). These results indicate that the difference in onset and offset FDI is not because of differences in response strength. Next, we tested whether the tendency for offsets to be less depressive than onsets was still found when response magnitude was measured as an AUC instead of a maximum FR, and again found that offset responses were less depressive than onset responses (Fig. 3*C*, FDI: −0.29 ± 0.50 vs −0.11 ± 0.47, Mann–Whitney *U* test: *p* < 0.001). Finally, we checked to see whether our results were dependent on cortical depth. Previous research has shown that history dependence is different across cortical depth, with deeper layers showing more onset depression than shallower layers (Christianson et al., 2011). We wondered whether offsets were also more depressive in the deeper layers. To determine whether cortical depth was significantly related to FDI, and whether this relationship differed for onset and offset FDIs, we binned the fractional depth into seven linearly spaced bins and performed a two-way ANOVA (depth bin vs onset/offset) (Fig. 3*D*). This test revealed a significant interaction between response type and cortical depth (*F*_(6, 2827)_ = 4.52, *p* < 0.001). *Post hoc* multiple comparisons tests confirmed that offsets were significantly less depressive than onsets for most of the cortical depth bins (5 of 7). This difference was not significant for two of the shallowest depth bins (the first and third shallowest depth bins), which is consistent with previous results (Christianson et al., 2011), that onset responses in superficial layers are less depressive than in deep layers.

In summary, these results show that, regardless of how they are measured or normalized, onset responses mainly depress in response to repeated sound stimulation, consistent with prior reports (Phillips et al., 2017; Seay et al., 2020). In contrast, and in addition to previous reports, offset responses are less likely to depress and more likely to either remain stable or facilitate.

### Within cells, the history dependence of onset and offset firing does not correlate

We found that ∼40% of cells in our sample responded both to sounds and to sound offsets. We wondered whether, in these cells, there was any correlation between the history dependence of the onset response and the offset response; this would be important for understanding how neurons downstream might decode the information they carry, and also for establishing whether the mechanisms underlying this history dependence are mainly presynaptic versus postsynaptic. Figure 4*A* shows the normalized PSTHs for all units showing both onset and offset responses, showing that, among units with onset and offset responses, onset responses were on average depressive and offset responses were on average stable, but that there was considerable variability in the short-term dynamics for both onset and offset responses. To quantify this difference and determine whether the cells with the most depressing onset responses also had the most depressing offset responses, we compared their FDIs. Within units, the offset FDI was significantly more positive than the onset FDI (Fig. 4*B*, right, −0.37 ± 0.54 vs −0.09 ± 0.46, Wilcoxon signed-rank test: *p* < 0.001). However, there was no tendency for units with larger onset FDIs to also have larger offset FDIs (Spearman's ρ: −0.05, *p* = 0.18, *n* = 849), and the offset FDI was not significantly different for units with very negative (<−0.2), intermediate (≥−0.2 and ≤0.2), or very positive (>0.2) onset FDIs (Fig. 4*C*, Kruskal–Wallis test: χ^2^ = 0.4, *p* = 0.81). The lack of a clear relationship between onset and offset FDIs within units can further be seen in Figure 4*D*, which shows an alluvial plot illustrating the relationship between cells showing very negative (depressive), intermediate (stable), or very positive (facilitatory) responses. Within cells, all three onset FDI profiles (depressive, stable, facilitatory) coexist with all three offset FDI profiles, and no dominant relationships are apparent. These results suggest that, over the time intervals used here, history dependence is mainly implemented presynaptically (independently for different ascending stimulus pathways), consistent with previous research, which shows that onset and offset responses are primarily driven by nonoverlapping sets of synapses (Scholl et al., 2010; Liu et al., 2019).

### Onset history dependence varies between cell types, but offset history dependence does not

*In vitro*, thalamocortical inputs to AC show cell type-specific short-term synaptic plasticity. For example, inputs driving PV cells typically depress, whereas inputs driving SST cells typically facilitate (Tan et al., 2008; Takesian et al., 2013). We therefore wondered whether the short-term dynamics of onset and offset responses *in vivo* would vary between cell types. We used opto-tagging to identify PV and SST cells in Pv-Cre;Ai32 and Sst-Cre;Ai32 mice (Fig. 5*A–E*). We additionally split the nontagged cells into BS and NS groups because prior work has shown that most BS cells are excitatory and nearly all NS cells are inhibitory (Barthó et al., 2004) (Fig. 5*E*).

We first determined the proportion of cells of each type showing onset and/or offset responses. Most PV cells responded both to sounds and to sound-offsets, and rarely responded to one but not the other (Fig. 6*Ai*). In contrast, SST cells typically responded only to sounds but not to sound-offsets (Fig. 6*Aii*), a result consistent with previous reports (Liu et al., 2019; Li et al., 2021). Some nontagged NS cells fired similarly to PV cells (i.e., firing to both sounds and sound-offsets), and others fired similarly to SST cells (i.e., firing to sounds but not to sound-offsets) (Fig. 6*Aiii*), consistent with the hypothesis that the NS cell population is a mixture of the PV and SST populations (for review, see Tremblay et al., 2016). Most cells in our recordings were broad spiking. These cells could show any combination of onset and offset responses, including “offset-only” and “neither” (i.e., sound-suppressed) firing (Fig. 6*Aiv*), patterns that were not observed among tagged or putative interneurons.

We next compared onset and offset response facilitation and depression across cell types. Figure 6*B* (left) shows the mean normalized PSTHs for the first and fourth onset response. For BS, NS, and PV cells, firing was typically transient and typically depressed between the first and fourth responses. In contrast, the firing of SST cells was typically more sustained and showed less depression. A Kruskal–Wallis test confirmed a significant effect of cell type on the onset FDI (Fig. 6*B*, right, Kruskal–Wallis test: χ^2^ = 11.9, *p* = 0.0076), and multiple comparisons tests revealed that SST cells were significantly less depressive than NS (Dunn's *post hoc* test: *p* = 0.0070) and BS cells (Dunn's *post hoc* test: *p* = 0.0059). In comparison, offset responses remained relatively stable for all cell types (Fig. 6*C*, left), and there were no significant differences between offset FDI for cell type (Fig. 6*C*, right, Kruskal–Wallis test: χ^2^ = 3.81, *p* = 0.28). Both SST and especially PV interneurons fire at much higher rates than pyramidal cells (Fig. 6*Di*, left, Kruskal–Wallis test: χ^2^ = 506.3, *p* < 0.001), a feature that may influence our ability to detect sound responses in their firing. To verify that the cell type differences in their likelihood of responding to sound are not simply because of cell type differences in FR, we subsampled the cell populations to create distributions matched for FR (Fig. 6*Di*, right). After this distribution matching procedure, the PV tagged cells still mainly responded to both sounds and sound-offsets, the SST tagged cells still responded mainly to sounds but not offsets, the NS cells showed both these patterns, and BS cells could respond to sounds or sound-offsets or both, indicating that these cell type characteristic response features did not depend wholly on FR (Fig. 6*Dii*). The major difference was that the BS “neither” (sound suppressed) cells were eliminated by this matching procedure because their maximal FRs were low or negative, a behavior that was almost never observed among tagged or putative interneurons. Cell type differences in onset and offset FDI were also largely unaffected by the matching procedure (Fig. 6*Diii*), indicating that these differences were not explained by FR.

### Early and late onset responses differ in their short-term dynamics, while early and late offset responses are similar to each other

The prior results show that onsets typically depress for all cell types, but that SST cells are the most likely to have stable or facilitative firing (i.e., their onset responses have slightly less negative FDIs than other cell types). However, the normalized PSTHs reveal a more complex picture, particularly for SST cells, whose firing early (0-50 ms) in the sound response appears generally depressive, but whose firing later in the sound response appears to facilitate (Fig. 6*B*, left, SST). We therefore measured their FDIs separately for firing in early (0-50 ms) and late (100-150 ms) onset response windows (Fig. 7*A*). Most SST cells fired in both the early and late response windows (Fig. 7*B*, left, Both category). This result is consistent with the normalized PSTHs presented in Figure 6*Aii*, which show that SST cells most often fired throughout the duration of the sound. Interestingly, SST cells' FDIs were drastically different for early and late firing, with early firing being typically depressive and late firing being typically facilitative (Fig. 7*B*, right, early firing FDI: −0.24 ± 0.60, late firing FDI: 0.25 ± 0.62). In comparison, BS and NS cells were much more likely to show early firing only (Fig. 7*C*, left), and the difference in their FDI for early versus late firing was less pronounced (Fig. 7*C*, middle and right, for BS cells: early firing FDI: −0.44 ± 0.53, late firing FDI: −0.08 ± 0.61; for NS cells: early firing FDI: −0.44 ± 0.36, late firing FDI: −0.03 ± 0.60), while PV cells almost never fired late in the response window at all (Fig. 7*C*, left). Across cell types, a two-way ANOVA with response window (early/late) as one factor and cell type (BS, NS, PV, SST) as another factor revealed a significant main effect of early versus late (*F*_(1, 2296)_ = 42.4, *p* < 0.001; the interaction between response window and cell type was nonsignificant: *F*_(3, 2294)_ = 1.01, *p* = 0.39), and Tukey *post hoc* tests revealed a significant effect of early versus late window for SST, NS, and BS cells (*p* < 0.001). In summary, early onset firing is typically depressive for all cell types, whereas late onset firing is stable (BS, NS), almost nonexistent (PV), or facilitatory (SST). In contrast to onset responses, offset history dependence did not differ significantly for early and late offset firing (Fig. 7*D*, two-way ANOVA: interaction between response window and cell type: *F*_(3, 1201)_ = 0.89, *p* = 0.45; simple main effects for early vs late: *F*_(1, 1203)_ = 0.01, *p* = 0.92).

### Offsets are more facilitatory than onsets, regardless of response duration

**Figure 8.**
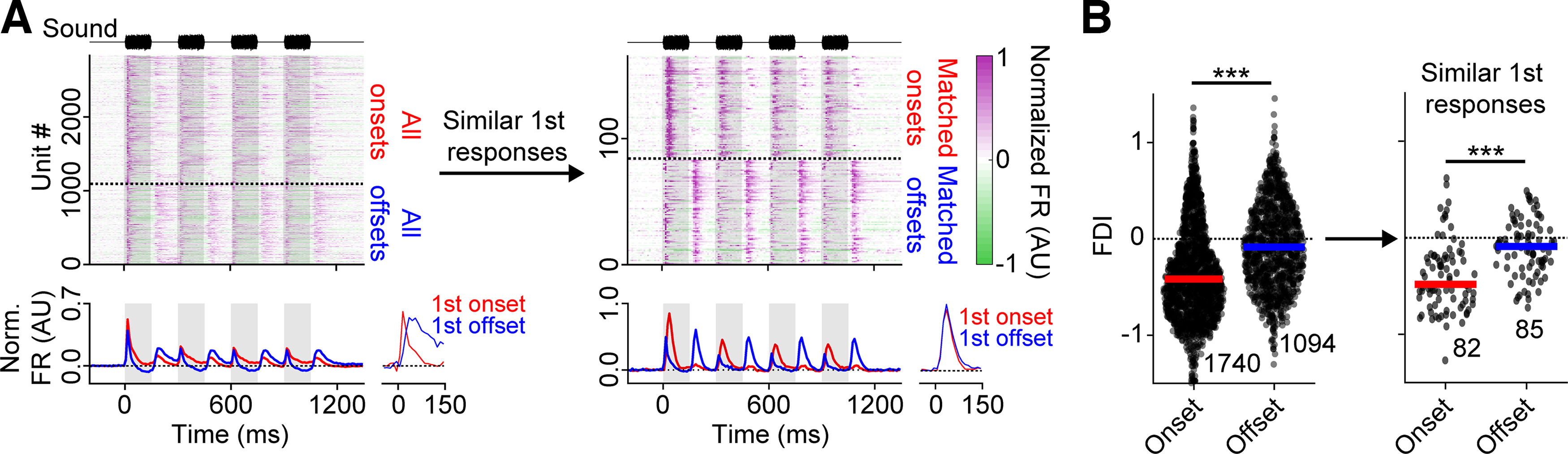
Offset responses are more facilitatory than onset responses, even among neurons whose response durations are similar. ***A***, Normalized PSTHs (top) and the mean of these normalized PSTHs (bottom) for units showing onset (red) and/or offset responses (blue). Left, All PSTHs with onset or offset responses. Right, PSTHs from cells whose first onset or offset responses match a defined template (see Materials and Methods). Insets, Mean initial responses rescaled and overlaid to facilitate comparing the dynamics of the two responses. ***B***, Onset and offset FDIs, for all cells with onset and offset responses (left, identical to Fig. 2*D*), and for matched cells only (right). In both cases, offset FDIs were significantly more positive than onset FDIs (all cells with onset or offset responses: *p* < 0.001, matched cells: *p* < 0.001). ***p < 0.001.

These results showed that, among onset responses, the earlier or more transient portions were more depressive, and the later or more sustained portions were comparatively facilitatory. Offset responses were generally less transient than onset responses (Figs. 1*F*, 6*B*,*C*), and were also more facilitatory than onset responses (Fig. 2*D*). Could offset responses' tendency to facilitate be explained simply by their more sustained firing? To test this possibility, we compared two subpopulations of cells: offset-responsive cells whose responses to the first sound offset were similar to one another and were transient (defined by a template matching procedure; see Materials and Methods), versus onset-responsive cells whose responses to the first sound onset had similar dynamics to the offset responses in the first group. Figure 8*A* shows the normalized PSTHs for all onset- and offset-responsive cells (left), and for the onset- and offset-responsive cells that were selected by this template matching procedure (right). Although across all cells in the population, the initial onset and offset responses were very different from one another (Fig. 8*A*, left, bottom, inset), for the cells selected by the matching procedure, the mean normalized initial onset and offset responses were almost identical (Fig. 8*A*, right, bottom, inset). Among these cells, offset FDIs were still significantly more positive than onset FDIs (Fig. 8*B*, right, −0.40 ± 0.36 vs −0.10 ± 0.29, Mann–Whitney *U* test: *p* < 0.001). We concluded that offset responses are more facilitatory than onset responses, and this cannot wholly be explained by differences in the transience of the response.

### Clustering cells based on firing throughout the stimulus reveals relationships with cell type and onset/offset response dynamics

**Figure 9.**
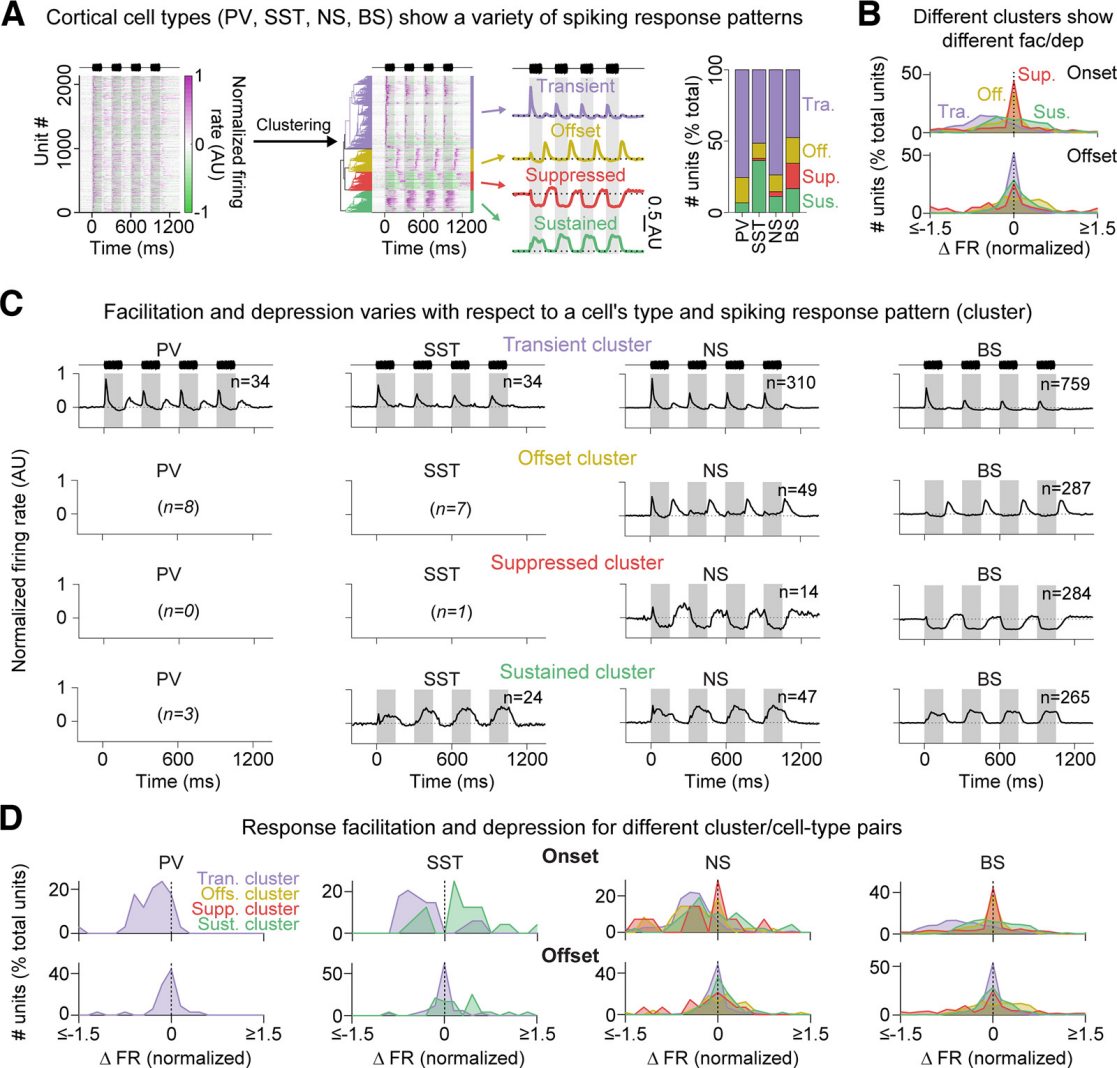
Clustering cortical cells based on their onset and offset spiking responses. ***A***, Normalized PSTHs (left) were clustered into four groups based on spiking response patterns (middle); PSTHs show the mean PSTH for each cluster. These four clusters showed (1) transient onset responses (Transient cluster, light purple), (2) stable offset responses (Offset cluster, yellow), (3) suppressed onset responses (Suppressed cluster, red), or (4) sustained onset responses (Sustained cluster, green). Stacked bar plots (right) show the proportion of cells that fell within each cluster, split according to cell type (PV, SST, NS, BS). ***B***, Histograms represent the change in normalized FR from the first to fourth response, for Clusters 1-4 (colors), and for onset (top) and offset (bottom) responses. **C**, ***D***, Mean normalized PSTHs (***C***) and the change in normalized FR histograms (***D***) for each cluster (1-4) and cell type (PV, SST, NS, BS) pair. Cluster/cell type pairs containing <10 cells are left blank.

The above results show that much of the cell-to-cell variation in short-term response dynamics could be explained by multiple factors, including cell type and the degree to which a cell fired transiently or sustained. It was also clear that these factors showed interesting relationships with one another. For example, PV cell onsets were generally transient and depressive, whereas SST cell onsets were more likely to be sustained and stable/facilitatory. To examine the interrelationships between these diverse response features, and to see which relationships were dominant in the cell population, we performed hierarchical clustering on all the normalized PSTHs (including those without clear onset or offset firing), using the PSTHs from the start of the first noise burst to 150 ms past the end of the last noise burst. We saw four main clusters (Fig. 9*A*), corresponding to cells with transient, depressing onset responses (Transient cluster, light purple), cells with stable offset responses (Offset cluster, yellow), cells with sustained onset responses (Sustained cluster, green), and cells with sound-suppressed firing (Suppressed cluster, red). Each cell type (BS, NS, PV, SST) contained cells from a variety of different clusters; thus, each cell type showed a variety of different spiking responses (Fig. 9*A*, right). Most PV cells fell into the transient/depressing cluster. This result is reflected in their normalized PSTHs (Fig. 6*Ai*), where most PV cells showed transient, depressive onset firing. Similar to PV cells, most NS cells fell within the transient/depressing onset cluster. This result is expected because most NS cells are PV cells (Keller et al., 2018). In contrast, although many SST cells fell into the transient/depressive cluster, others fell into the sustained/stable cluster. This result is also reflected in their normalized PSTHs (Fig. 6*Aii*), where many SST cells show either transient or sustained firing, and also in their onset FDIs, which showed a roughly bimodal distribution (Figs. 6*B*, 7). Many BS cells were found in each of the four clusters, and interestingly, nearly all sound-suppressed cells were broad-spiking.

To compare response dynamics between clusters, we measured the change in normalized FR from the first to fourth response (taking the extremal value in each response window). For this analysis, we did not use the FDI to measure response dynamics because the FDI measures changes in maximal FR, and does not capture the dynamics of response suppression. For onset responses (Fig. 9*B*, top), cells in the Transient cluster showed depressive firing, whereas cells in Sustained cluster fired stably. Cells in the Suppressed cluster typically showed no change in FR across the stimulus repetitions, indicating that sound-suppressive firing is generally neither facilitatory nor depressive. Cells in the Offset cluster did not generally fire to sound onsets. For offset responses (Fig. 9*B*, bottom), the cells in the Offset cluster tended to slightly facilitate, which is in accordance with our previous results (Fig. 2*D*). Small offset responses were also observed in the Suppressed cluster, although it was hard to determine whether they depressed or facilitated. This was because of sound-suppressed responses frequently lingering into the offset response windows, which erroneously resulted in negative FRs for offset responses, and this inflated the number of offset responses in this cluster showing depression (Fig. 9*B*, bottom, red, count of units below 0).

To determine how cell type, spiking response patterns (cluster), and short-term response dynamics interact, we calculated the normalized PSTHs and FR changes for each cluster/cell type pair (Fig. 9*C*,*D*). Within clusters, cell types typically manifested the cluster's spiking response pattern but with subtle cell type specific variations: for instance, BS cells in the Transient cluster were typically onset-only (Fig. 9*C*, top right), but NS and PV cells in this same cluster typically had weak offset responses as well (e.g., Fig. 9*C*, top left and top right, middle). These changes also manifested in response dynamics (Fig. 9*D*). For instance, BS cells in the Transient cluster (Fig. 9*D*, top right, light purple) were more strongly depressed than PV (Fig. 9*D*, top left, light purple) or SST (Fig. 9*D*, top left middle, light purple) cells in the same cluster. The BS cells in the Sustained cluster showed stable firing (Fig. 9*D*, top right, green), and the SST cells in the Sustained cluster were more likely to facilitate (Fig. 9*D*, top left middle, green). Interestingly, SST response dynamics were highly dependent on cluster identity: SST cells in the Transient cluster were nearly always depressive (Fig. 9*D*, light purple), whereas SST cells in the Sustained cluster were mostly facilitative (Fig. 9*D*, green). These and previous results (Figs. 6, 7) suggest that SST cells fall into two broad categories. We wondered whether these two groups of SST cells showed different depth distributions, but they did not (fractional depth for transient SST cells: 0.62 ± 0.21, and for sustained SST cells: 0.59 ± 0.17, Mann–Whitney *U* test: *p* = 0.56). In summary, these results show that cells within the AC show considerable diversity in their onset and offset response dynamics, and this diversity relates to each cell's type and its spiking response pattern.

## Discussion

We show that, like onset responses, offset responses change depending on the recent history of sound presentation. Whereas onset responses typically depressed or remained stable, offset responses were less likely to depress and more likely to facilitate, and this finding was robust to a variety of analysis choices and controls. Among neurons showing both onset and offset responses, the sign and magnitude of a neuron's onset history dependence did not predict the neuron's offset history dependence. Excitatory neurons and PV interneurons are much more likely to fire offset responses than SST neurons; but among the cells that do fire offset responses, all cell types are equally likely to have those offset responses facilitate or depress. While onset responses showed a clear relationship between response duration and facilitation/depression (slower responses were more likely to facilitate), offset responses were more facilitatory than could be explained by their duration. Last, responses to our noise burst stimulus generally fell into broad clusters which vary in the presence, duration, and sign of onset or offset responses. Cell types differ in how likely they are to belong to one or another cluster; and even within a cluster, different cell types show their own characteristic response dynamics.

### Why might offset responses depress less than onset responses?

The tendency for offset responses to depress less than onset responses may be inherited from upstream sites, shaped through intracortical or thalamocortical mechanisms, or both. Offset responses are observed throughout the central auditory system (Scholl et al., 2010; Kopp-Scheinpflug et al., 2018). However, it is not known whether offset responses in subcortical structures themselves tend to facilitate or tend to depress. In either case, because the thalamocortical synapses that carry information about sound offsets are separate from those carrying information about sound onsets (Scholl et al., 2010; Liu et al., 2019), the onset and offset inputs to AC from the thalamus may be differentially filtered by presynaptic mechanisms regardless of whether they show different response dynamics in the medial geniculate body (Markram et al., 1998). Intracortical mechanisms, including excitatory-inhibitory interactions, may also be important. Among these, our data point to an intriguing possibility regarding SST interneurons. Prior work has shown that these cells contribute to the depression of onset firing, such that their inactivation decreases the proportion of cortical cells showing onset depression (Phillips et al., 2017). Very few SST cells fire offset responses (Fig. 6), consistent with prior work (Liu et al., 2019; Li et al., 2021). If SST-mediated inhibition actively shapes onset responses but these cells do not fire during the offset response windows, this absence of a key mechanism producing onset depression could partly explain why offsets are less depressive. Future optogenetic experiments or modeling could explore this possibility. An additional possibility is that cell-intrinsic ionic mechanisms shape or even generate offset responses; for instance, akin to the situation in the superior paraolivary nucleus, where postinhibitory rebound allows superior paraolivary nucleus cells to transform ascending inhibition into offset responses (Kopp-Scheinpflug et al., 2011) and these mechanisms contribute to offset dynamics. Indeed, recent work by Sołyga and Barkat (2021) has suggested that offset responses may be generated *de novo* in the AC, thus suggesting that offset history dependence may also be generated *de novo* in the AC. However, if cell-to-cell variation in cell-intrinsic ionic mechanisms is responsible for the difference between onset and offset history dependence in the AC, we would expect that, among neurons that show both onset and offset responses, we would see correlations between the short-term dynamics of the onset and offset responses. The absence of such correlations suggests that cell-to-cell variation in intrinsic properties is probably not a major explanatory factor.

### Cell type differences in the prevalence of onset and offset responses

In accordance with previous findings, untagged BS cells (mainly excitatory), untagged NS cells (mainly inhibitory), and opto-tagged PV cells frequently showed both onset and offset firing, whereas SST cells were more likely to show onset firing only (Liu et al., 2019; Li et al., 2021). Offset responses are likely more difficult to detect in SST cells, in which the onset firing frequently lasted throughout the duration of the sound presentation, which in turn could have masked offset responses; however, both visual inspection and clustering of PSTHs supported the conclusion that SST cells rarely show offset responses (Figs. 6, 9).

### Cell type differences in history dependence

SST cells were also unique in showing less onset depression (Figs. 6*B*, 7*B*,*C*). *In vitro*, thalamocortical inputs driving PV cells are mostly depressing, whereas the thalamocortical inputs driving SST cells are mostly facilitatory, both in somatosensory cortex (Tan et al., 2008) and in AC (Takesian et al., 2013). Our data are generally consistent with these prior results; and yet, if the thalamocortical inputs driving SST cells are almost invariably facilitative (Takesian et al., 2013), why do our results show heterogeneity in their short-term dynamics? There is a complex array of intracortical connections between different cell types, which contribute to sensory responses (Kepecs and Fishell, 2014; Kato et al., 2017; Wood et al., 2017), and which show various forms of short-term synaptic plasticity (Reyes et al., 1998; Beierlein et al., 2003). In contrast to *in vitro* preparations, these connections will remain relatively intact in the *in vivo* condition, and these will undoubtedly increase variability in results. In contrast to onset firing, there were no cell type-specific differences in offset history dependence. It is interesting to speculate regarding the relative importance of precise temporal information for the functions served by onset and offset responses, and the relative importance of processing by intracortical circuitry versus long-range transmission of information.

### Response patterns and history dependence

We observed many different patterns of responses in AC (e.g., transient, suppressed, sustained), and these were related to a cell's tendency to show depression or facilitation. For onset responses, early (0-50 ms) firing was nearly always more depressive than late (100-150 ms) firing (Fig. 7*C*, right). In a study comparing transient and sustained firing in the AC, Wang et al. (2005) showed that “preferred” stimuli (at a neuron's best modulation frequency) are more likely to evoke sustained firing, whereas “nonpreferred” stimuli (away from a neuron's best modulation frequency) are more likely to evoke transient firing. This raises two possibilities: first, the cells that we classified as transient/depressing may have shown more sustained/stable firing in response to different test stimuli (e.g., to sustained best-frequency pure tones) consistent with our inference that response facilitation or depression depends on the dynamics of the specific synapses carrying the information, or the specific cortical circuitry modulating it, but not the biophysical properties of the particular neuron being recorded. Second, history dependence may aid this process, such that depressive firing will further limit responses to nonpreferred stimuli.

We also observed cell type-specific differences in spiking response patterns. The most notable difference was a tendency for PV cells to fire transiently in response to sounds, while SST cells tended to fire in a more sustained manner. These results are consistent with our expectation that cells with depressive thalamocortical inputs (e.g., PV) will likely fire in a transient/depressive manner, whereas cells with facilitative thalamocortical inputs (e.g., SST) will likely fire in a sustained/stable manner. We also noted that some BS cells, and almost no other cells, showed suppression of spontaneous firing in response to sound. This is opposite to the sustained response pattern observed in SST cells, which often directly inhibit BS cells (Silberberg and Markram, 2007; Urban-Ciecko and Barth, 2016). Future work (e.g., optogenetic experiments suppressing SST cells) might establish whether SST to BS connections are responsible for this response suppression. In contrast, we observed little diversity among offset responses; for example, suppressed or sustained offset firing was almost never seen (Fig. 9), and facilitation/depression indices were similar for early and late offset firing (Fig. 7*D*). Future studies will be needed to determine how onset and offset responses are interpreted in later stages of neural processing, and why such diverse forms of onset responses, but not offset responses, are required.

This work shows that offset responses are sensitive to sound history. It will now be interesting to determine the role of this history dependence in hearing. Li et al. (2021) have shown that an increase in offset response magnitude aids in the discrimination of tone duration. This leads to the question: if offset responses are subject to depression and facilitation, how can sound duration encoding function stably in different sound contexts? Similarly, does this history dependence affect the encoding of other aspects of sound in which offset responses are essential, such as FM sweeps and silent gaps, which are themselves crucial for the understanding of complex, continuous sounds, such as speech, and consequently the integration with other senses required for communication? Up until now, the study of offset responses has mainly used individual sound stimuli isolated in time (i.e., tones or noise bursts with long intertrial intervals). This work shows that temporal aspects must also be considered if we are to fully understand the function of offset responses in hearing.
